# Complete Genome Sequence of a Natural Recombinant H9N2 Influenza Virus Isolated from a White-Fronted Goose (*Anser albifrons*) in South Korea

**DOI:** 10.1128/genomeA.00149-13

**Published:** 2013-05-23

**Authors:** Dong-Hun Lee, Jae-Keun Park, Seong-Su Yuk, Tseren-Ochir Erdene-Ochir, Jung-Hoon Kwon, Joong-Bok Lee, Seung-Yong Park, In-Soo Choi, Chang-Seon Song

**Affiliations:** Avian Disease Laboratory, College of Veterinary Medicine, Konkuk University, Gwangjin-gu, Seoul, Republic of Korea

## Abstract

In 2007, we isolated a natural recombinant H9N2 avian influenza virus (AIV) from the fecal droppings of a white-fronted goose (*Anser albifrons*) in South Korea. Phylogenetic analyses of the complete genome sequence showed that polymerase acidic (PA) and neuraminidase (NA) genes belonged to the Eurasian lineage AIV, but polymerase basic 2 (PB2), PB1, hemagglutinin (HA), nucleoprotein (NP), matrix (M), and nonstructural (NS) genes belonged to the North-American lineage AIV. These data are beneficial for understanding the ecology and epidemiology of AIVs.

## GENOME ANNOUNCEMENT

Avian influenza virus (AIV) belongs to the *Influenzavirus A* genus of the *Orthomyxoviridae* family (1). The AIV genome consists of eight single-stranded negative-sense segments, including polymerase basic 2 (PB2), PB1, polymerase acidic (PA), hemagglutinin (HA), nucleoprotein (NP), neuraminidase (NA), matrix (M), and nonstructural (NS) genes. AIV is classified into subtypes based on the antigenic differences between their two surface glycoproteins, HA and NA. AIV can be genetically distinguished by geographical origin: North American and Eurasian. In a previous study, North American-Eurasian reassortant virus subtypes H3N8 and H4N6 were detected during AIV surveillance of live-animal market and wild-bird habitats in South Korea (2).

In the present study, an H9N2 strain named A/white-fronted goose/Korea/20-36/2007 (H9N2) was isolated from the fecal droppings of a white-fronted goose (*Anser albifrons*) in South Korea in 2007. Species identification of the fecal droppings was done by DNA barcoding using the cytochrome oxidase I gene, as described previously (3). The complete genome of the virus was amplified by reverse transcription-PCR and sequenced with an ABI Prism 3730xl genetic analyzer (Applied Biosystems, Foster City, CA). The results indicated that the lengths of each segment for PB2, PB1, PA, HA, NP, NA, M, and NS, were 2,296, 2,309, 2,165, 1,683, 1,502, 1,410, 989, and 857 nucleotides, respectively. The eight genes encoded the following proteins, followed by the deduced amino acid lengths: PB2, 759; PB1, 757; PB1-F2, 90; PA, 716; HA, 560; NP, 498; NA, 469; M1, 252; M2, 97; NS1, 230; and NS2, 121. The deduced amino acids at the cleavage site of the HA protein were PAASDR↓GLF, with the characteristic of low pathogenic property.

Phylogenetic analysis revealed that the PB2, PB1, HA, NP, M, and NS genes of A/white-fronted goose/Korea/20-36/2007 (H9N2) virus were derived from North American lineages but that PA and NA genes were derived from Eurasian lineages. It seems that continuous reshuffling of AIV genes between North American and Eurasian lineages has been occurring in wild bird populations, producing the reassortant North American-Eurasian lineage AIV.

In the present study, our results provide crucial information on the epidemiology of AIV in wild birds by increasing understanding of the role of migratory birds in exchanging AIV genes between the Eurasian and North American continents. Enhanced surveillance of the wild bird population is required to improve our understanding of the natural history of AIV in wild birds.

### Nucleotide sequence accession numbers.

The genome sequences of A/white-fronted goose/Korea/20-36/2007 (H9N2) have been deposited in GenBank under accession no. KC693639 to KC693646.
